# RNA-binding motif protein 10 represses tumor progression through the Wnt/β- catenin pathway in lung adenocarcinoma

**DOI:** 10.7150/ijbs.63598

**Published:** 2022-01-01

**Authors:** Yingyue Cao, Jianxiong Geng, Xin Wang, Qingwei Meng, Shanqi Xu, Yaoguo Lang, Yongxu Zhou, Lishuang Qi, Zijie Wang, Zixin Wei, Yan Yu, Shi Jin, Bo Pan

**Affiliations:** 1 Department of Medical Oncology, Harbin Medical University Cancer Hospital, Haping Road No 150, Harbin 150040, China.; 2 Department of hepatopancreatobiliary surgery, Second Affiliated Hospital of Harbin Medical University, Harbin, 150040, China.; 3 College of Bioinformatics Science and Technology, Harbin Medical University, Harbin, 150040, China.; 4 National Cancer Center/National Clinical Research Center for Cancer/Cancer Hospital & Shenzhen Hospital, Chinese Academy of Medical Sciences and Perking Union Medical College, Shenzhen, 518116, China.

**Keywords:** RBM10, progression, Wnt/β-catenin pathway, CTNNBIP1, lung adenocarcinoma

## Abstract

RNA-binding motif protein 10 (RBM10), one of the members of the RNA-binding protein (RBP) family, has a tumor suppressor role in multiple cancers. However, the functional role of RBM10 in lung adenocarcinoma (LUAD) and the underlying molecular mechanism remains unclear. In this study, we observed that RBM10 is significantly downregulated in LUAD tissues compared with normal tissues. Low RBM10 expression is significantly associated with poor outcome of LUAD patients. *In vitro* and* in vivo* experiments show that RBM10 inhibits cell proliferation, metastasis and EMT progression in LUAD. Mechanistically, we demonstrate that RBM10 interacts with β-catenin interacting protein 1 (CTNNBIP1) and positively regulates its expression, disrupting the binding of β-catenin to the transcription factor TCF/LEF, thereby inactivating the Wnt/β-catenin pathway. In conclusion, this is the first study reporting the role of RBM10 in suppressing LUAD progression at least via partly inactivating the Wnt/β-catenin pathway, which provides new insights into the tumorigenesis and metastasis of LUAD. Thus, RBM10 may be a promising new therapeutic target or clinical biomarker for LUAD therapy in the future.

## Introduction

Lung cancer is the second most frequently diagnosed cancer in the world and is the leading cause of cancer-related mortality worldwide 1. In 2020, 2.2 million new cases (11.4%) and 1.8 million deaths (18%) occurred worldwide 2. Lung adenocarcinoma (LUAD) represents the most frequent histological type of lung cancer. Despite rapid advances in cancer diagnosis and treatment, the 5-year survival rate for LUAD patients remain lower than 15% 3. Thus, identifying new therapeutic targets for LUAD is crucial importance.

RNA-binding motif protein 10 (RBM10), also known as S1-1, is often deleted or mutated in malignant cells 4-6, including LUAD 7, 8. The major biological functions of RBM10 include regulation of mRNA stabilization, alternative splicing, nuclear output, and translation 9-11; yet, the exact role of RBM10 in cancer progression remains controversial. Loss of RBM10 can promote cell proliferation, migration, and invasion in osteosarcoma 12. Furthermore, RBM10 can promote the BAX expression in breast cancer, which suggests that RBM10 is a potential tumor suppressor 13. However, other studies suggested that RBM10 might have a pro-cancer role as a tumor promoter or a pro-oncogene 9, 14-16. In invasive melanoma, higher RBM10 expression was positively correlated with increased disease aggression 17. RBM10 expression contributes to tumor growth and metastasis in RBM5-null tumors 14. In our previous study, we performed second-generation sequencing of samples collected from 19 patients with LUAD with metastasis and found that RBM10 was downregulated in LUAD. Moreover, low expression of RBM10 was associated with late clinical stage and poor prognosis of lung adenocarcinoma patients. Therefore, we hypothesized that RBM10, as a tumor suppressor gene, may be involved in LUAD progression.

Epithelial-mesenchymal transition (EMT) is considered a key indicator in the initial step of cancer metastasis 18. EMT is a complex process that involves multiple signaling pathways, including the Wnt/β-catenin pathway, PI3K/AKT pathway, TGF-β pathway, and MAPK pathway 18. A Wnt/β-catenin pathway is one of the most important signaling pathways, whose dysregulation is often seen in malignant cells, including LUAD 19-21. The Wnt/β-catenin pathway is an important regulator of EMT, its activation facilitates EMT to promote invasion and metastasis of various tumors 22, 23. CTNNBIP1 (also known as ICAT), is one of the β-catenin negative regulatory factors, which binds to β-catenin and prevents the interaction between β-catenin and the TCF/LEF complex. This inactivates the transcription of target genes downstream of the Wnt pathway, thereby inhibiting the activation of the Wnt/β-catenin pathway 24. Therefore, it is often used as an inhibitor of the Wnt/β-catenin pathway. Previous studies have reported that CTNNBIP1 is involved in the progression of various tumors, including malignant melanoma 25, glioblastoma 26, colorectal cancer 27, and cervical cancer 28. In lung cancer, ectopic expression of CTNNBIP1 can inhibit cell migration, while its down-regulation can cause an opposite effect 29. However, so far, no study has reported on the relationship between RBM10 and CTNNBIP1 in LUAD.

The aim of this study was to determine whether RBM10 could suppress LUAD progress and metastasis by regulating EMT via the Wnt/β-catenin signaling pathway.

## Materials and Methods

### Bioinformatics analysis

The Oncomine (http://www.oncomine.org) and GEPIA (http://gepia.cancer-pku.cn/) were used to analyze the mRNA expression level of RBM10 in LUAD tissues and the normal lung tissues. The Kaplan-Meier plotter (http://kmplot.com) database was used to analyze the correlation between RBM10 expression and survival prognosis in patients with LUAD.

### Human fresh LUAD tissues

Six paired fresh samples, including LUAD tumor and adjacent normal lung tissues, were collected from the Third Clinical Thoracic Surgery Department, Harbin Medical University, according to clear pathological diagnosis and patient informed consent. All procedures were approved by the Third Clinical Ethics Committee of Harbin Medical University (the ethical permission number: KY2019-19).

### Cell culture

Human LUAD cell lines H1299, H1915, H1650, A549, H1975, H661, H827, and PC-9, and the normal lung epithelial cell line HBE were all obtained from American Type Culture Collection (ATCC). All culture media were supplemented with 10% fetal bovine serum (FBS, PAN, Biotech GmbH, Germany). PC-9 was cultured in Dulbecco's Modified Eagle's Medium (DMEM, Gibco^®^, Grand Island, NY, USA), while the remaining cell lines were cultured in RPMI-1640 (Gibco^®^) at 37 °C in a humidified atmosphere containing 5% CO2. Cells used in experiments were in good condition without mycoplasma contamination.

### Cell Transfection

The small-interfering RNA (siRNA) targeting RBM10 and CTNNBIP1 were purchased from Ribobio (Guangzhou, China). The used siRNA sequences were: RBM10 siRNA-1, GCATGACTATGACGACTCA; RBM10 siRNA-3, CGACGGACATAAGGAGACA, and CTNNBIP1, siRNA-1, 5'-GAUGGGAUCAAACCUGACA-3′. A negative siRNA control (si-NC) with the sequence 5'-UUCUCCGAACGUGUCACGUTT-3' was also used.

A549 and H1299 cell lines were cultured on a 6-well plate for 24 h. Cells were then using 10 μl of the required siRNA (50 μM) together with 10 μl jet-PRIME (Poly-plus Transfection, France) according to the manufacturer's instructions. The design of the overexpression RBM10 sequence and the packaging of lentiviruses were completed by the Han bio Biotechnology Company (Shanghai, China). A549 and H1299 cell lines were infected with lentivirus using polybrene (6 μg/ml) and then selected with puromycin (2 μg/ml) for 14 days to establish the stable RBM10-overexpressing cell lines. The transfection efficiencies were verified by qRT-PCR and western blot.

### Quantitative real-time PCR (qRT-qPCR) analysis

Total RNA was isolated from LUAD cells using TRIzol reagent (Invitrogen, Carlsbad, CA, USA) according to the manufacturer's instructions. The cDNA was obtained through reverse transcription with a Fast Quant RT Kit (TIANGEN, China). GAPDH was used as the internal control. qRT-PCR was conducted on a 7500 fast PCR System (Applied Biosystems, Foster City, CA, USA) using Talent qPCR Pre-Mix (SYBR Green; TIANGEN, China). The specific mRNA expression level was quantified using the 2-ΔΔCT method. The primers utilized in qRT-PCR are listed in **Table S1**. Each experiment was run in triplicate.

### Western blot

The standard Western blot experiment was performed as previously described 30, using 60 μg protein samples from fresh tissues and cells. The antibodies used for the Western blot analysis are listed in **Table S2**. Each experiment was run in triplicate.

### Cell proliferation assays

Cells were counted and seeded in 96-well plates (5 **×** 10^3^ cells/well). After incubation for 24 h, 10 μl of Cell Counting Kit-8 (CCK-8, Dojindo, Kumamoto, Japan) was added to the culture medium and incubated for 1.5 h at 37 °C. Then, the optical density (OD) value at 450 nm was measured by using SpectraMax Paradigm (Molecular Device, CA, USA). All of the values were standardized by comparison with the data from the untreated cells. Three independent experiments were performed.

### Clone formation assay

Transfected cells (700 cells/well) were counted and plated in 6-well plates. After 14 days of culture, cells were fixed with 0.4% paraformaldehyde for 15 min and were then stained with 0.5% crystal violet for 30 min. Colonies containing more than 50 cells were counted. All of the experiments were replicated 3 times.

### 5-Ethynyl-2′-deoxyuridine (EdU) incorporation assay

After transfection, the LUAD cells were inoculated into 24-well plates. EdU kit (RiboBio, Guangzhou, China) was used for labeling cells following the manufacturer's instructions. Photographs were taken using an inverted fluorescent microscope (Leica Microsystems Inc., USA), and the experiment was repeated three times.

### Soft agar colony formation assay

Soft agar colony formation assay (GENMED SCIENTIFICS INC, USA) was performed according to manufacturer's instructions. Briefly, 1.5mL GENMED Cloning Solution (Reagent A) and 1.5mL GENMED Hypertrophic Solution (Reagent B) were mixed and added into the 12-well plate, after which the substrate was solidified. Next, 1ml GENMED aqueous reagent (Reagent C) with 500ul GENMED clonal reagent (Reagent A) and 200μl cell suspension (containing 2500 cells) were mixed and immediately added into the 12-well plate. The colloid was set at room temperature for 2h and incubated overnight at 37 °C and 5% CO_2_. The next day, 1mL GENMED Reagent D was added into the 12-well plate and cultured at 37 °C and 5% CO_2_ for 4 weeks. Photographs were taken under an inverted microscope (Leica Microsystems Inc., USA). Three independent experiments were performed.

### Wound healing assay

After transfection, the A549 and H1299 cells were seeded into 6-well plates. When the cell density reached over 80%, a 200μl pipette tip was used to scratch three separate wounds through the cells, moving perpendicular to the line. The cells were then gently rinsed twice with PBS to remove floating cells and cultured in the medium containing 0.5% FBS serum for 48 hours. Images of the scratches were taken using an inverted microscope (Olympus, Tokyo, Japan) at ×10 magnification at 0 and 48 h of incubation. The experiments were run in triplicate.

### Transwell assay

In brief, 3~5×10^5^ cells were resuspended in 300ul serum-free medium and then seeded in the upper chamber (BD Biosciences, New Jersey, USA) pre-coated with or without 40 µl diluted Matrigel, while 700 µl medium supplemented with 10% FBS was added in the lower chamber. After 24 h or 48 h, cells on the top surface of the microporous membrane were wiped off with a cotton swab. The remaining cells were fixed with 4% paraformaldehyde, stained with 0.1% crystal violet, and counted per 3 random fields for each assay under a microscope (Leica Microsystems Inc., USA). The data are obtained from three independent experiments.

### FITC-phalloidine cytoskeleton staining and cell immunofluorescence staining

Cells were slightly washed by preheated (37 °C) PBS 3 times and fixed in 4% paraformaldehyde for 20 min. Then, the cells were permeated with 0.5% Triton X-100 for 5 min and blocked in 5% BSA for 1h at room temperature. For FITC-phalloidine cytoskeleton staining, F-actin was stained with TRITC (SolarBio, Beijing, China) containing 1% BSA for 40 min at room temperature. For cell IF staining, the cells were incubated with the rabbit polyclonal anti-E-cadherin antibody (diluted 1:100) and the rabbit polyclonal anti-Vimentin antibody (diluted 1:100) primary antibodies at 4 °C overnight. The next day, the relevant secondary antibodies were added to the above cells for 1h at room temperature. The nuclei were stained with DAPI for 5~8min. The cells were imaged using an inverted fluorescence microscope (Leica Microsystems Inc., USA).

### TOP/FOP flash reporter assay

A549 and H1299 with stable RBM10 overexpression were cultured in 24-well plates (2 × 10^4^ cells per well). After 24 h, cells were transfected with the TOP-Flash or FOP-Flash reporter plasmids together with pRL-TK using Lipofectamine 2000 (Invitrogen). After 48h of culture, the luciferase activity was analyzed using a dual-luciferase reporter kit (Promega). Data are presented as the ratio of relative light units of TOP flash to FOP flash from triplicate experiments.

### Nuclear and cytoplasmic protein extraction

Cytosolic and nuclear protein extraction was performed using a Minute^TM^ Cytoplasmic and Nuclear Extraction Kit for Cells (Invent, SC-003) according to the manufacturer's instructions. In brief, the cells were washed twice with cold PBS, after which the buffer was completely aspirated. Cells were then mixed with an appropriate amount of cytoplasmic extraction buffer and placed on ice for 5 min, centrifuged for 5 min at 14,000×g at 4 °C, after which the supernatant was collected (cytosol fraction). Next, samples were mixed with an appropriate amount of nuclear extraction buffer to pellet, vigorously vortexing for 60 seconds, and then incubated on ice for 15min; this procedure was repeated 4 times, after which samples were centrifuged for 2 min at 14,000×g. Each fraction was tested for the presence of the cytosolic marker β-actin and the nuclear marker laminB1 by Western blotting as appropriate. Each experiment was performed three times.

### Co-immunoprecipitation (Co-IP)

Co-immunoprecipitation was conducted according to manufacturer's operations (Absin Bioscience *Inc*, china). Briefly, the cells were washed three times with ice-cold PBS. The cell lysate was then collected at 4 °C using immunoprecipitation lysis buffer supplemented with protease inhibitor (Roche, Basel, Switzerland). The 500 μl of cell lysates (containing total protein 200-1000 ug) were precleared with 5 μl of protein A and protein G agarose beads at 4 °C for 2h. Then, the cell lysates (500 μL) were incubated with 5 μg of the antibody and 1 ug of the normal IgG antibody at 4 °C overnight. The next day, samples were mixed with an immunoprecipitation mixture (5 μl of protein A and protein G beads) for 3h. The immune-complex was collected, washed 6 times with cold IP buffer by a 2 min centrifugation at 12,000×*g.* Samples were analyzed by Western blotting. Each experiment was performed three times.

### Chemicals

XAV-939 (a specific inhibitor of Wnt/β-catenin signaling) and CHIR-99021 (a specific activator of Wnt/β-catenin signaling) were purchased from Selleckchem. All agents were used according to the manufacturers' instructions.

### Animal experiments

Female nude mice (BALB/c, 4 weeks) were purchased from Beijing Vital Li Hua Experimental Animal Technology Company (Beijing, China). The animal experiments were performed under a project license (NO.:KY2017-12) granted by the Third Clinical Ethics Committee of Harbin Medical University. All animal studies were undertaken in compliance with the regulations and guidelines of Harbin Medical University institutional animal care. For xenograft model construction, 2.5 × 10^7^/150 μl A549 cells with or without stable RBM10 overexpression (A549-Vector or A549-RBM10) were subcutaneously injected into 4 weeks BALB**/**c nude mice (n=5 mice per group). The length and width of tumors were measured every 4 days with a caliper, and the tumor volume (mm^3^) was calculated with the formula: V=0.5×(length)×(width)^2^. The progression of xenograft growth was analyzed on day 32 using *in vivo* imaging system, after which the mice were sacrificed, the tumor dissected, weighed, and fixed in formalin. For lung metastasis models, the same female nude mice (n=5 mice per group) were injected with 1 × 10^7^/150μl A549-Vecoter or A549-RBM10 cells via the tail vein. The mice were sacrificed after 7 weeks, after which the lungs were excised and then analyzed in *ex vivo* using bioluminescence imaging (BLI) and hematoxylin and eosin (H&E) staining.

### IHC and H&E staining

IHC was performed as previously described 30. For primary antibody incubation, ki67 (27309-1-AP, Proteintech, 1:100), E-cadherin (20874-1-AP, Proteintech, 1:1000), Vimentin (10366-1-AP, Proteintech, 1:1000), c-MYC (CY5150, Abways, 1:40) and cyclinD1 (CY5404, Abways, 1:40) were used for IHC. For H&E staining, after dewaxing and rehydrating, longitudinal sections of 5μm were stained with hematoxylin solution for 5min, then soaked in 1% acidic ethanol (1% HCl in 70% ethanol) for 5 times, and finally rinsed in distilled water. The sections were then stained in eosin solution for 3min, then dehydrated with gradient alcohol, and clarified in xylene. Eventually, a microscope (Olympus, Toyo, Japan) was used to observe the tissue sections.

### Statistical analysis

All experiments were independently repeated at 3 times. Statistical analysis was performed with GraphPad Prism 6.0 software (San Diego, California, USA). All data were shown as the mean ± SD, unless declared. Data were analyzed using Student's t-test for two groups or one-way analysis of variance (ANOVA) for three or more groups. A *P* value < 0.05 was considered to be statistically significant.

## Results

### Low RBM10 expression is associated with a poor prognosis in LUAD

**Figure 1:**
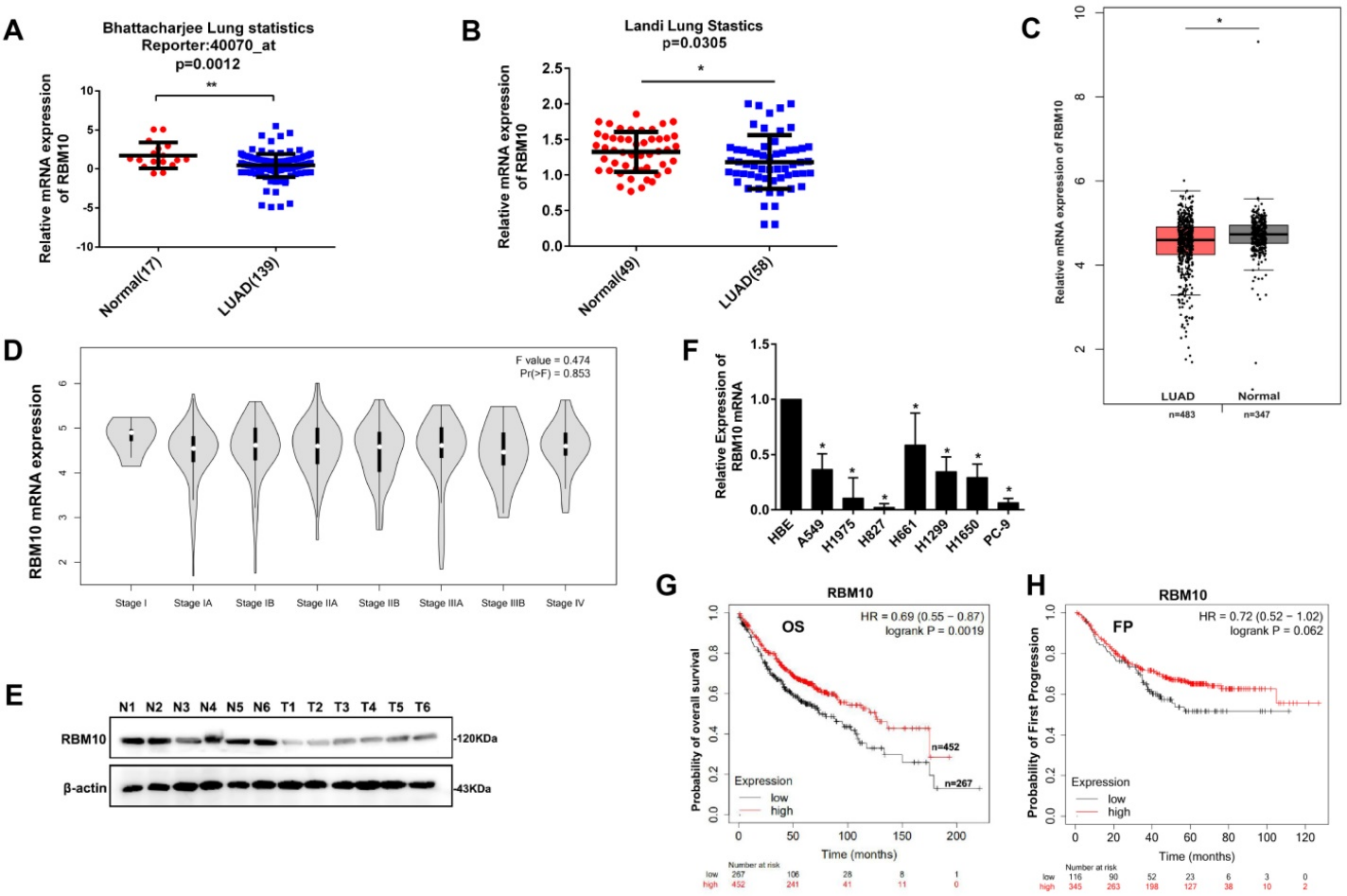
** Low RBM10 expression is associated with a poor prognosis in LUAD. (A, B)** RBM10 mRNA levels of LUAD compared with the normal lung sample in Bhattacharjee lung cohort (A) and Landi Lung cohort (B) based on Oncomine statistics (http://www.oncomine.org). **(C)** The boxplot analysis showed the RBM10 mRNA expression level in LUAD from TCGA and GTEx database on the GEPIA website (http://gepia.cancer-pku.cn/). **(D)** The box plots showed that expression levels of RBM10 were gradually decreased with clinical stage progression. **(E)** Western blot analysis of the protein expression of RBM10 in sex pairs of human LUAD fresh tissues. **(F)** qRT-PCR results of the mRNA expression of RBM10 in eight LUAD cell lines (H1299, H1915, H1650, A549, H1975, H661, H827, PC-9) and the normal lung epithelial cell line HBE. **(G)** Kaplan-Meier plotter database (http://kmplot.com) was searched for the overall survival of LUAD patients. **(H)** Kaplan-Meier plotter database (http://kmplot.com) was searched for the First Progression (FP) of LUAD patients.

Based on Bhattacharjee lung and Landi Lung cohort from Oncomine database (http://www.oncomine.org), RBM10 mRNA expression was downregulated in LUAD tissues compared with corresponding normal lung tissues (p<0.05, **Figure 1A, B**). Furthermore, the RBM10 mRNA expression was low in LUAD and gradually decreased with clinical stage progression from TCGA and GTEx database in GEPIA website (http://gepia.cancer-pku.cn/) (**Figure 1C, D**). Moreover, western blot analysis showed that RBM10 protein expression was downregulated in human LUAD fresh tissues (**Figure 1E**). Consistently, the results of qRT-PCR analysis also showed that RBM10 mRNA expression was lower in LUAD cell lines compared with the normal lung epithelial cell line HBE (**Figure 1F**). In addition, the Kaplan-Meier plotter database (http://kmplot.com) analysis showed that patients with low RBM10 expression had poor overall survival (OS) compared to patients with high RBM10 expression (HR = 0.72, Log-rank P = 0.0068, **Figure 1G**). We also found that the patients with low RBM10 expression had poor first progression (FP) compared to patients with high RBM10 expression in LUAD (HR= 0.72, Log-rank P= 0.062, **Figure 1H**), although not statistically significant. Taken together, these results indicated that RBM10 may be a suppressor gene in LUAD.

### RBM10 inhibits cell proliferation, migration, and invasion of LUAD cells *in vitro*

**Figure 2:**
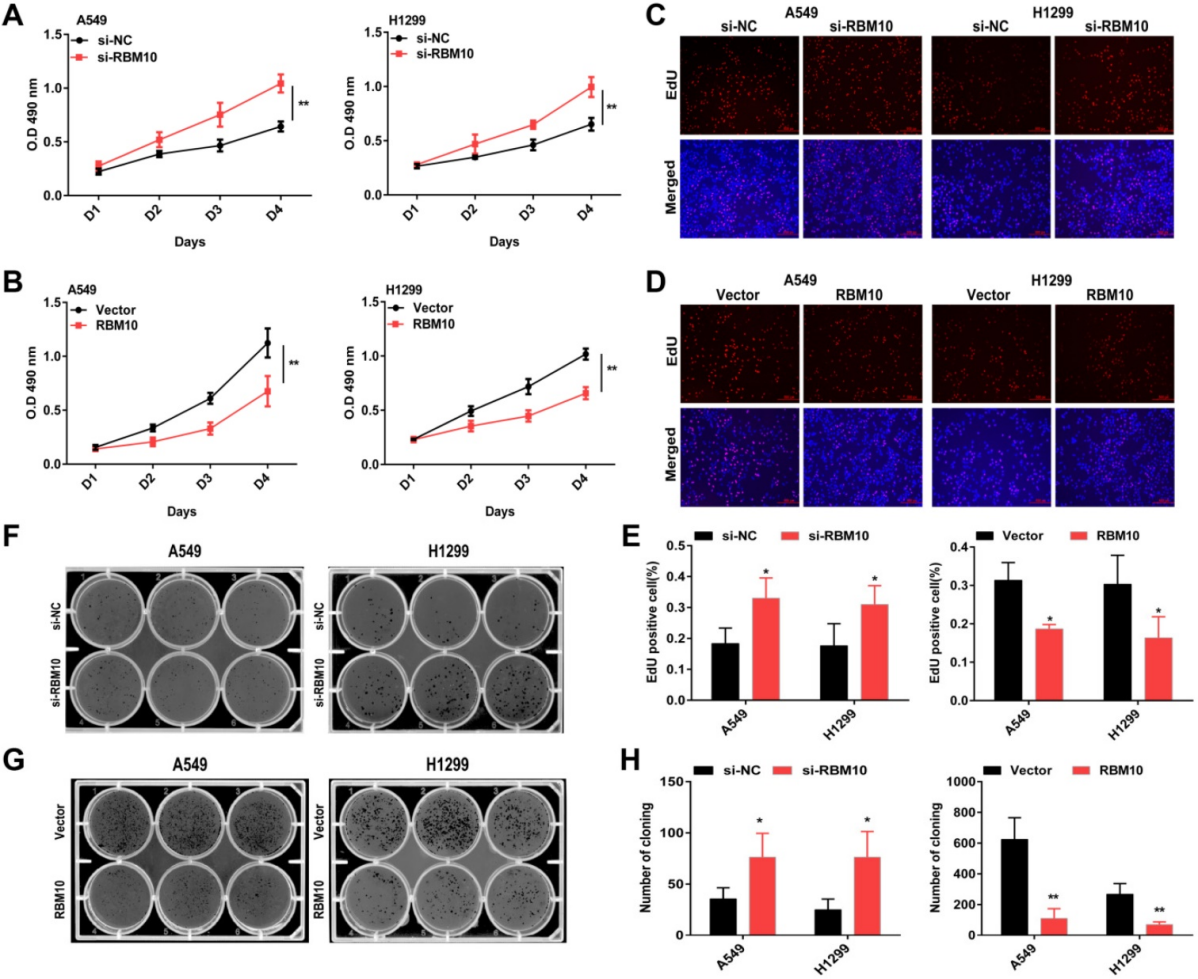
** RBM10 inhibits cell proliferation of LUAD cells *in vitro.*** RBM10 was silenced or overexpressed in A549 and H1299 cell lines.** (A, B)** Cell proliferation was examined by CCK8; **(C-E)** EdU assays (scale bar is 100 µm, cells synthesizing DNA stained with EDU (red), Nuclei counter stained with Hoechst 33342 (blue) and **(F-H)** colony formation assays in A549 and H1299 cells. The results were represented as mean ± SD, *P < 0.05, **P < 0.01. Each experiment was repeated three times.

**Figure 3:**
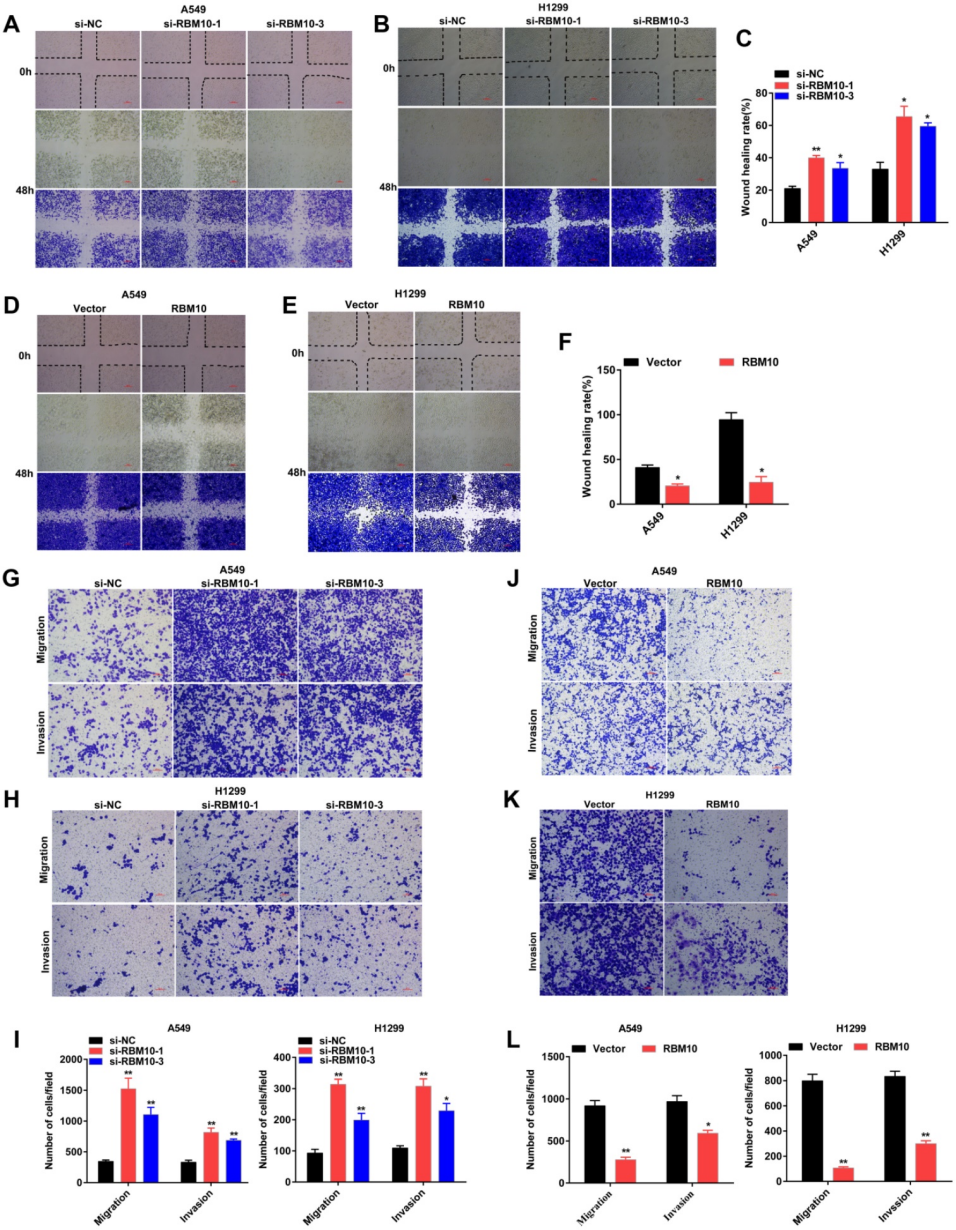
** RBM10 inhibits cell migration and invasion of LUAD cells *in vitro.*** RBM10 was silenced or overexpressed in A549 and H1299 cell lines. **(A-F)** A wound healing assay was used to test the migration capacity of RBM10 in LUAD cells. The cells migrating into the wounded areas were photographed at 0h and 48h. The results were represented as mean ± SD, *P < 0.05, **P < 0.01, Scale bar is 100 µm. **(G-L)** The migration and invasion capacity of RBM10 in LUAD cells were also examined by Transwell assays. The results were represented as mean ± SD, *P < 0.05, **P < 0.01, Scale bar is 100 µm. Each experiment was repeated three times.

The above data showed that A549 and H1299 cells have a moderate RBM10 expression level (**Figure 1F**). A549 and H1299 cell lines are known to be the most aggressive and malignant. Based on these results, we silenced RBM10 in A549 and H1299 cells with RBM10-siRNA to knock down RBM10 expression. At the same time, RBM10 was stably overexpressed using an RBM10 lentivirus in same LUAD cells. Both the overexpression and knockdown efficiencies of RBM10 were confirmed by qRT-PCR and Western blot assays (**Figure S1A-D**). We then performed a variety of *in vitro* experiments to evaluate the effect of RBM10 expression on cell proliferation, migration, and invasion of LUAD cells**.** The CCK-8 and EdU assays showed that RBM10 knockdown significantly increased cell viability and enhanced the DNA synthesis ability of both A549 and H1299 cells (**Figure 2A, C, E**), while forced RBM10 expression caused an opposite effect (**Figure 2B-E**). Moreover, the inhibition of RBM10 led to the generation of more and larger cell colonies compared with the control groups (**Figure 2F, H**), while overexpressing RBM10 reduced both colony size and number in LUAD cells (**Figure 2G, H**). These findings were further confirmed in soft agar colony formation assays (**Figure S1E, F**). We also found that RBM10 silencing promoted the invasion and migration ability of A549 and H1299 cells, whereas RBM10-overexpressing LUAD cells reduced the cell invasion and migration capacity by wound-healing assays (**Figure 3A-F**) and Transwell assays (**Figure 3G-L**). Thus, these results suggest that RBM10 may inhibit tumor cell proliferation, migration, and invasion of LUAD cells *in vitro*.

### RBM10 suppresses LUAD cell tumorigenesis and metastasis *in vivo*

**Figure 4:**
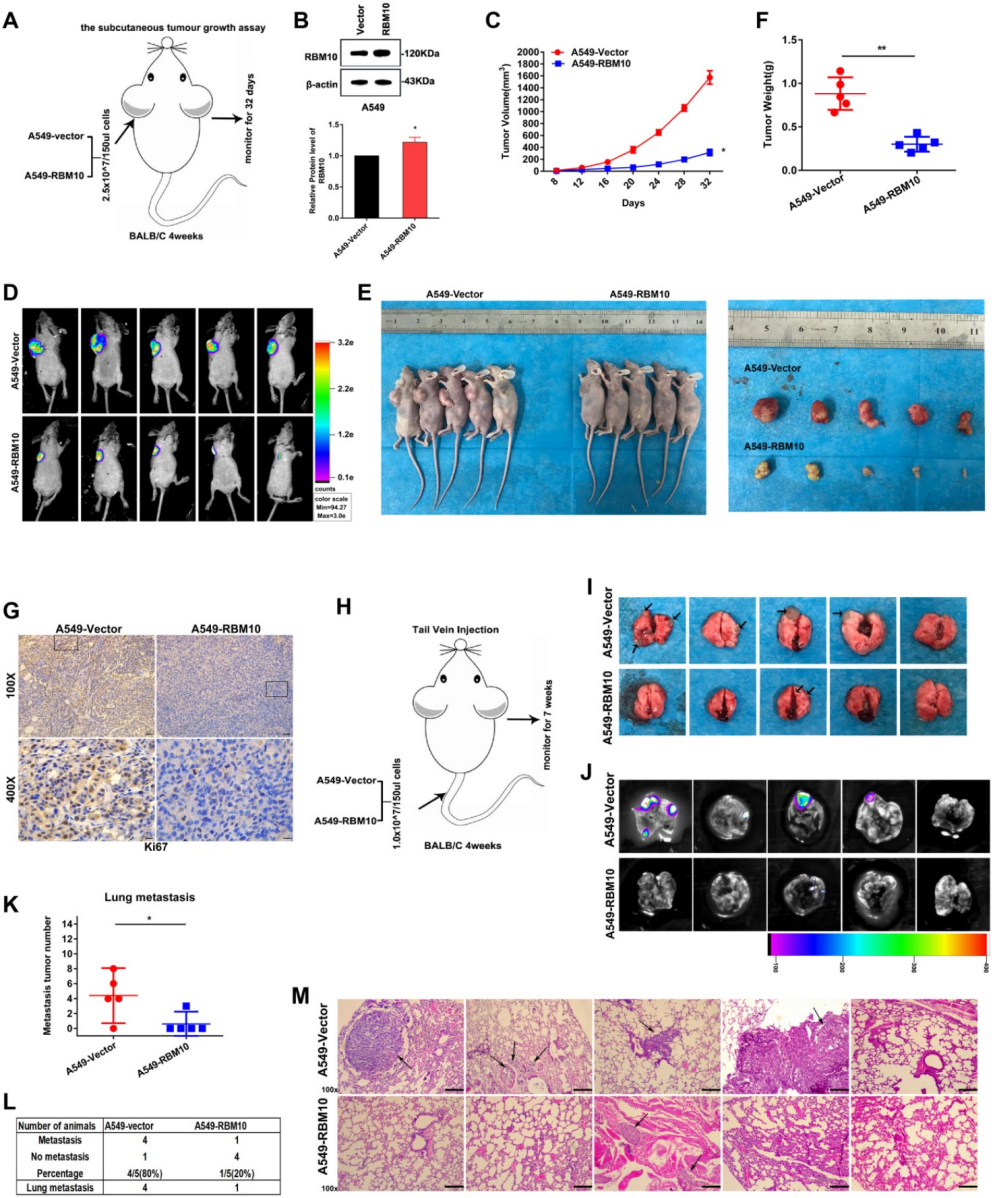
** RBM10 suppresses LUAD cell tumorigenesis and metastasis *in vivo*.** Approximately 2.5 × 10^7^/150μl A549 cells expressing RBM10 were injected into the left armpit of 4 weeks BALB/c nude mice (n=5 mice per group); tumor volume was measured after sizeable tumors formation (day 8), and measured every 4 days. On day 32, the inoculated mice were sacrificed, photographed, and the tumor was weighted. **(A)** Schematic diagram of subcutaneous xenograft model in BALB/c nude mice**. (B)** Stable overexpression of RBM10 in A549 cells and the transfection efficiency were assessed by Western blot. **(C)** The subcutaneous tumor volume curves. **(D)** Representative images of bioluminescence imaging (BLI) of the nude mice 32 days after injection of indicated cells. **(E)** Representative gross photos of mice and the tumor lumps from the indicated groups at the endpoint of the experiment. **(F)** Tumor weight was measured. **(G)** The expression of ki67 in tumor tissues was determined by IHC, Scale bar is 100μm. The results were represented as mean ± SD, *p<0.05, **p<0.01. A total of 1 × 10^7^/150 µl stable RBM10 overexpression A549 cells were injected in nude mice through the tail vein to establish lung metastasis models (n=5 mice per group). **(H)** Schematic diagram of lung metastasis model in BALB/c nude mice. **(I, J)** After week 7, after tail vein injection, the lungs of mice were removed, **(I)** Representative gross images of the lungs from different experimental groups,** (J)** typical bioluminescence images of lung metastases; arrows indicate metastatic surface nodules. **(K)** Box-scatter plot shows the number of metastatic nodules in the lung as observed in each group. The results were represented as mean ± SD, *p<0.05.** (L)** Statistical analysis of lung metastasis in the two groups of nude mice. **(M)** Typical H&E staining pictures of lung metastasis lesions were shown. Arrows indicate metastatic surface nodules.

Next, we explored that RBM10 suppressed LUAD tumorigenesis and metastasis *in vivo*. A xenograft tumor mouse model was established by subcutaneously injecting 2.5 × 10^7^/150μl A549-Vector and A549-RBM10 cells into the left armpit of 4 weeks BALB**/**c nude mice (**Figure 4A**). Western blot assay showed that RBM10 was stably overexpressed in A549 cell lines (**Figure 4B**). As shown in **Figure 4C**, tumor volumes significantly decreased in the A549-RBM10 group compared with the A549-vector group. At the end of the experiments, the mice were sacrificed, the subcutaneous tumors were isolated, and their volume and weights were measured. The results showed that tumor volumes of the subcutaneous tumor tissues were markedly decreased in the A549-RBM10 group when compared with the A549-Vector group (**Figure 4D-F**, *P*<0.05). Furthermore, IHC analyses showed that the xenograft tumors from the A549-RBM10 group displayed a lower level of Ki67 relative to control (A549-Vector) (**Figure 4G**).

We also used the tail vein injection mouse model to investigate the influence of RBM10 in LUAD metastasis *in vivo* (**Figure 4H**). On day 49 after inoculation, the mice were sacrificed, the lungs were collected, and metastatic nodules were counted. The results indicated that the number and the size of lung metastasis lesions were significantly decreased in mice injected with A549-RBM10 cells relative to control (A549-Vector) cells (**Figure 4I-K,** *p*<0.05). Moreover, fewer mice in the A549-RBM10 group (1/5, 20%) showed lung metastasis, while in the control group (A549-Vector), almost mice developed lung metastasis (4/5, 80%, **Figure 4L**). In addition, we also found that the A549-RBM10 group had smaller and fewer lung metastatic foci than those in the control group (A549-vector) (**Figure 4M**). Collectively, these data indicated that RBM10 overexpression inhibited LUAD lung metastasis *in vivo*.

### RBM10 inhibits the EMT program of LUAD

**Figure 5:**
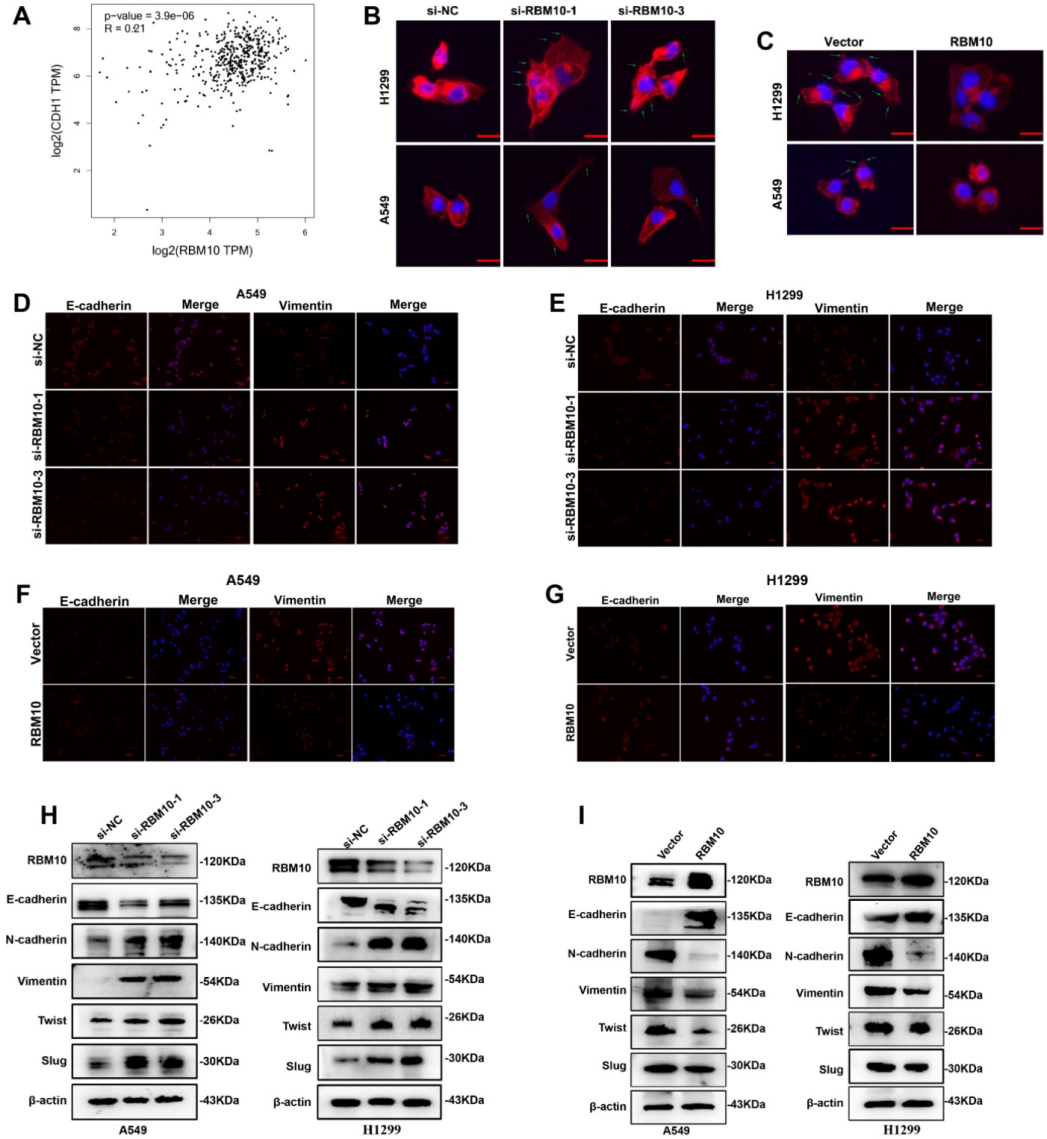
** RBM10 inhibits the EMT program of LUAD. (A)** Positive correlation between RBM10 and CDH1(E-cadherin) in LUAD, analyzed at the GEPIA website. **(B, C)** Rearrangements of the cytoskeleton (red) in A549 and H1299 cells when RBM10 was up- or down-regulated. Nuclei counter stained with DAPI (blue). **(D-G)** Immunofluorescence analysis used to observe the expression of epithelial cell markers E-cadherin (red) and mesenchymal cell markers vimentin (red) in A549 and NCI-H1299 cells with knockdown (D, E) or over-expressing (F, G) RBM10. Nuclei counter stained with DAPI (blue). **(H, I)** Western blot analysis of the EMT markers (E-cadherin, Vimentin, slug, twist, N-cadherin) in A549 or NCI-H1299 cells with knockdown (H) or overexpressing (I) RBM10.

Through LUAD-TCGA database, we found that RBM10 mRNA expression is positively correlated with CHD1 (also called E-cadherin) but negatively correlated with Vimentin (VIM), ZEB1, and ZEB2 (**Figure 5A** and **Figure S2**), indicating that RBM10 might participate in EMT process of LUAD. The cytoskeleton can trigger microfilament structural changes and increase the number of pseudopodia (lamellipodia and filopodia), which is responsible for cancer cells' invasive and migratory properties 31. The results of FITC-phalloidine cytoskeleton staining showed that RBM10-silencing cells formed a large number of visible actin filaments and pseudopodia compared with control cells, while RBM10-overexpressing cells had clear and round cell shapes bearing scarcely actin remodeling (**Figure 5B, C**). Using IF assays, we observed that the fluorescence intensity of E-cadherin decreased and Vimentin increased in the RBM10 silencing cells (**Figure 5D, E**). These effects were reversed by RBM10 overexpression (**Figure 5F, G**). A similar result was also revealed by IHC in tumor tissues from xenograft tumors (**Figure S3A**). Western blot analysis demonstrated that RBM10 silencing increased Vimentin, N-cadherin, slug, and twist protein expression levels, whereas E-cadherin protein expression levels were decreased in LUAD cells (**Figure 5H**). Conversely, overexpression of RBM10 showed the opposite effect (**Figure 5I**). Additionally, we also evaluated the mRNA expression of E-cadherin, Vimentin, slug, and twist in LUAD cells, and changes of mRNA expression were consistent with that observed at the protein level (**Figure S3B, C**). Furthermore, qRT-PCR assays showed that downregulation of RBM10 clearly increased the mRNA levels of ZEB1, ZEB2, MMP3, MMP7, and MMP10, while upregulation of RBM10 markedly reduced their mRNA expression (**Figure S3D-F**). Thus, these results strongly suggested that RBM10 inhibited EMT.

### RBM10 negatively regulates the Wnt/β-catenin pathway

To elucidate the underlying molecular mechanisms through which RBM10 regulates LUAD progression, the RNA sequencing (RNA-seq) was performed using H1299 cells that express either control si-NC or si-RBM10. Gene ontology (GO) enrichment analysis revealed that RBM10-dependent genes were involved in either biological processes such as biological adhesion, cell proliferation, and growth, or cellular component, including cell junction (**Figure S4A**), supporting a role for RBM10 in cell proliferation and EMT. KEGG pathway analysis showed that these genes were significantly associated with cancer-related functions, including cellular motility, growth and death and etc. (**Figure S4B**). More importantly, pathway enrichment analysis suggested that multiple signaling pathways might participate in the tumor-promoting mechanism of silencing RBM10, such as the Wnt**/**β-catenin pathway, NF-KB pathway, TGF-β pathway (**Figure S4C**). Based on StarBase databases, we found that the expression of RBM10 was markedly negatively correlated with the three common Wnt**/**β-catenin pathway target genes such as CTNNB1 (also called β-catenin), Wnt5a, and CD44 in LUAD (**Figure S5A**). Hence, Wnt/β-catenin pathway was selected for further research.

**Figure 6:**
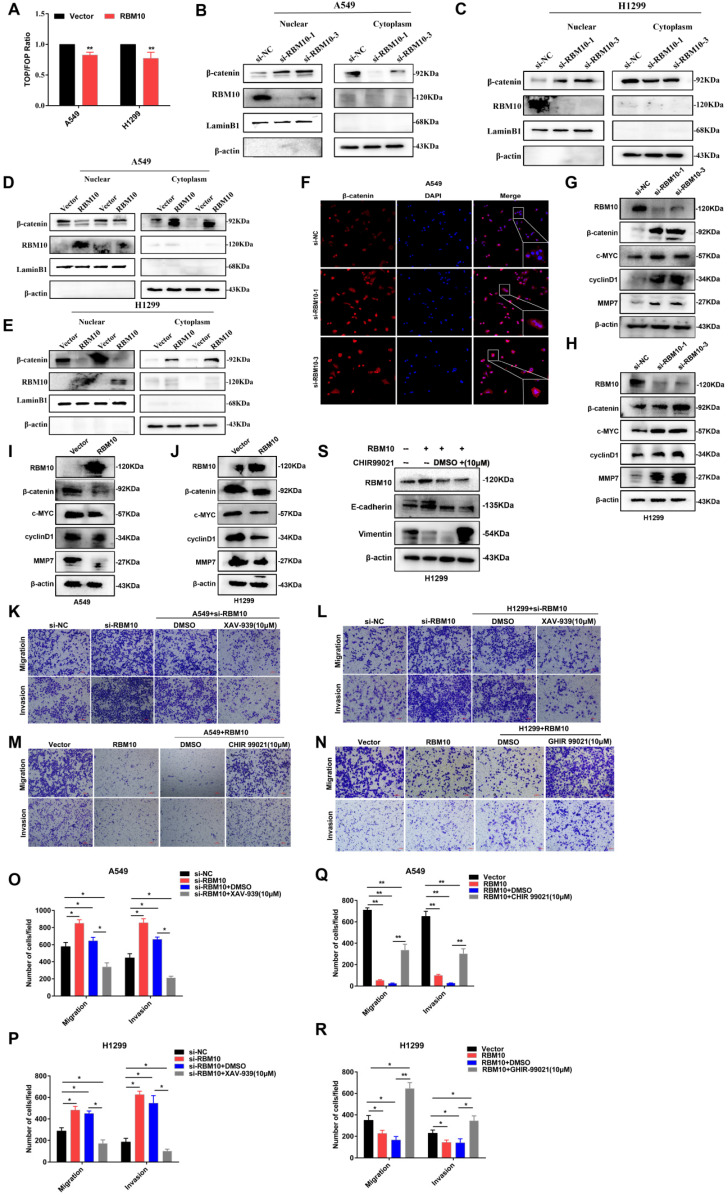
** RBM10 negatively regulates the Wnt/β-catenin signaling pathway. (A)** The TOP/FOP reporter assay was used to examine the activity of the Wnt/β‐catenin pathway in RBM10‐overexpressing A549 and H1299 cells. **(B-E)** Western blot assay nuclear and cytoplasmic cellular fractions utilized to detect the nuclear translocation of β-catenin. β-actin was the cytoplasmic control, and LaminB1 was the nuclear control. **(F)** Subcellular localization of β-catenin (red) in RBM10-silencing A549 cells was detected by immunofluorescence assay. Nuclei counter stained with DAPI (blue). **(G-J)** β-catenin, cyclinD1, c-MYC, and MMP7 levels were detected by Western blot in LUAD cells with RBM10 knockdown (G, H) or overexpression (I, J) respectively. **(K, L, O, P)** A Wnt/β-catenin pathway inhibitor, XAV‐939 (10 µM), was used to treat the RBM10‐silencing cells and the control cells for 48 h. The invasive and migration abilities of A549 and H1299 cells were tested by Transwell assay. **(M, N, Q, R)** A Wnt/β-catenin pathway activator, CHIR 99021 (10 µM), was used to treat the RBM10‐overexpressing cells and the control cells for 48 h. The invasive and migration abilities of LUAD cells (A549 and H1299) were evaluated by Transwell assay. **(S)** The protein levels of E-cadherin and Vimentin of H1299-RBM10 cells treated with CHIR 99021 (10 µM) was determined using western blot. The results were represented as mean ± SD. *p<0.05, **p<0.01. All experiments were repeated three times.

TOP**/**FOP flash luciferase reporter assays showed that RBM10 overexpression significantly reduced the activity of the TOP**/**FOP‐flash reporter genes in LUAD cells compared to control cells (**Figure 6A**), suggesting that RBM10 inhibits the Wnt**/**β-catenin pathway activity in LUAD cells. It has reported that that the key events of classical Wnt pathway activation are the stabilization and nuclear translocation of β-catenin. The nuclear and cytoplasmic protein extraction and Western blot assays results showed that the level of β-catenin in the nucleus was increased, while that in the cytoplasm was decreased by silencing RBM10 (**Figure 6B, C**). In contrast, overexpressing RBM10 made impaired nuclear β-catenin and induced cytoplasmic β-catenin (**Figure 6D, E**). Moreover, the subcellular localization of β-catenin in A549 cells detected by immunofluorescence (IF) analysis further supported our hypothesis (**Figure 6F and Figure S5D**).

We also examined the expression of c-MYC, cyclin D1, and MMP7, which are important downstream target genes of the Wnt**/**β-catenin pathway 32, 33. As shown in **Figure 6G-J** and **Figure S5B, C**, the results indicated that the mRNA and protein expression levels of β-catenin, c-MYC, MMP7, and cyclinD1 were up- or down-regulated when RBM10 was silenced or overexpressed in A549 and H1299 cells. In addition, IHC staining showed that c-MYC and cyclinD1 expression was lower in xenograft tumors with RBM10 overexpression (**Figure S5E**).

To further investigate the functions of Wnt/β-catenin pathway on the progression of LUAD, cells were treated with Wnt/β-catenin pathway activator CHIR 99021[34]or inhibitor XAV939^35^. As shown in **Figure 6K, L, O and P**, when cells were treated with XAV-939 (10μM) for 24h, Transwell assays indicated that the migration and invasion ability of A549 and H1299 RBM10 silencing cells was significantly decreased. However, CHIR 99021 (10 μM) promoted the migration and invasion ability of RBM10-overexpressing cells (**Figure 6M, N, Q and R**). Furthermore, Western blot showed that CHIR 99021 reverses the effect of RBM10 overexpressed on EMT markers (E-cadherin and Vimentin) expression (**Figure 6S**). Altogether, all these data indicated that depletion of RBM10 might promote LUAD cell proliferation and metastasis through promoting the activation of the Wnt**/**β-catenin pathway.

### RBM10 interacts with CTNNBIP1

**Figure 7:**
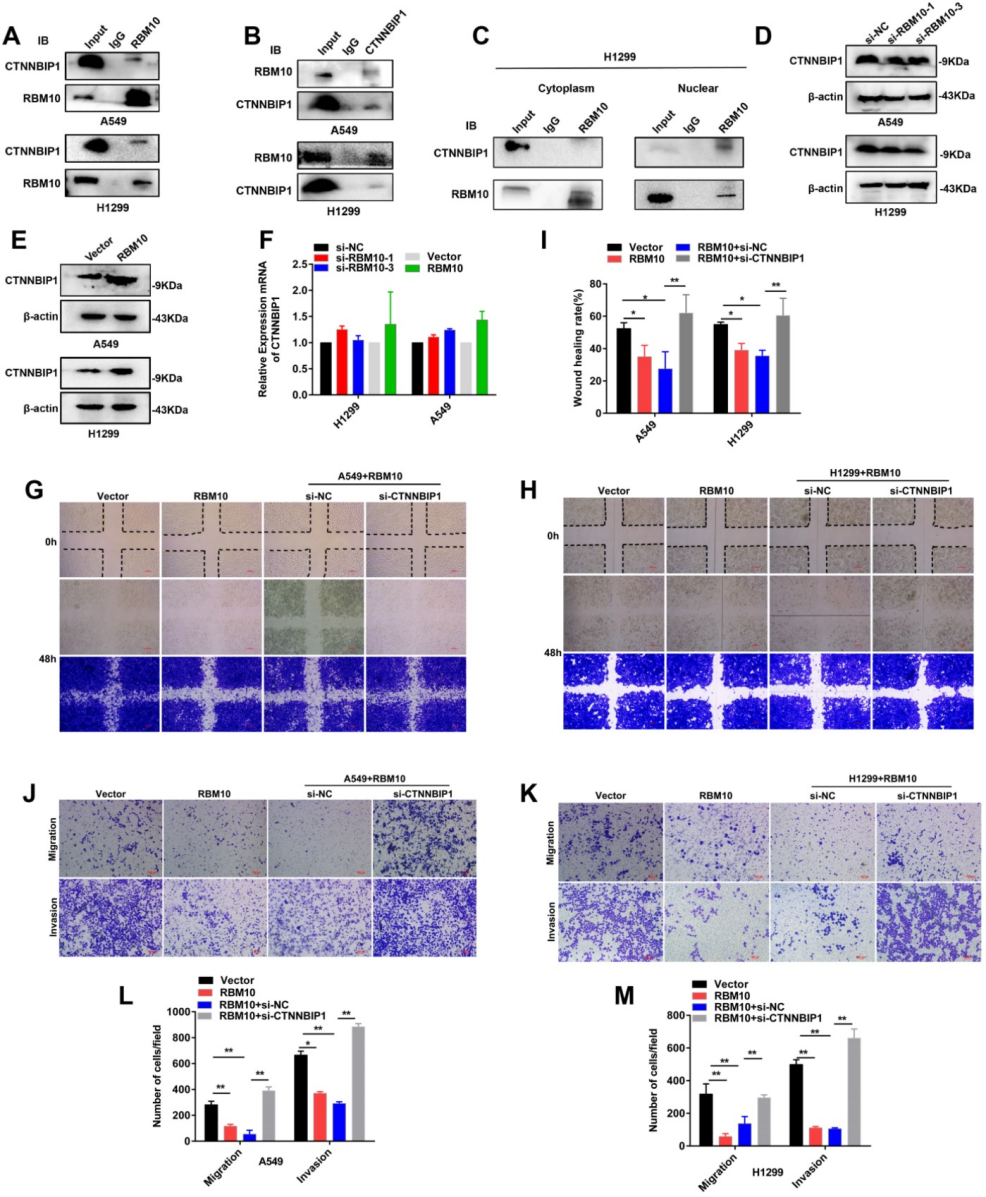
** RBM10 interacts with CTNNBIP1. (A, B)** Co-immunoprecipitation (Co-IP) was used to validate the interaction of RBM10 and CTNNBIP1, RBM10 was pulled down by anti-RBM10, and Western blot was used to detect RBM10 and CTNNBIP1; CTNNBIP1 was pulled down by anti-CTNNBIP1, and then RBM10 and CTNNBIP1 were detected by western blot. **(C)** co-IP assay of the nuclear and cytoplasmic cellular fractions revealed that RBM10 mainly interacted with CTNNBIP1 in the nucleus. **(D, E)** Western blot assay was utilized to detect RBM10 and CTNNBIP1 expressions in RBM10 knockdown or overexpression cells. β-actin was used as loading controls. **(F)** qTR-PCR result of RBM10 and CTNNBIP1 mRNA expressions under RBM10 knockdown or overexpression. Both down-regulation or upregulation of RBM10 did not alter CTNNBIP1 mRNA. GAPDH was used as loading controls. The results were represented as mean ± SD, *P < 0.05. **(G~M)** After transfection with si-CTNNBIP1 in stable RBM10 for 48 h, the cell migration, and invasion ability were estimated by wound-healing assays (**G-I**) and Transwell assays (**J-M**). The results were represented as mean ± SD, Scale bar is 100 µm *P < 0.05, **P<0.01. All experiments were repeated three times.

Co-immunoprecipitation (co-IP) experiments showed that RBM10 interacts with CTNNBIP1 (**Figure 7A, B**). Furthermore, co-IP assay of the nuclear and cytoplasmic cellular fractions showed that RBM10 mainly interacted with CTNNBIP1 in the nucleus (**Figure 7C**). Furthermore, we also observed that RBM10 silencing decreased the CTNNBIP1 protein level in LUAD cells, while RBM10-overexpression caused an opposite effect (**Figure 7D, E**). Down-regulation or upregulation of RBM10 did not significantly alter the levels of CTNNBIP1 mRNA transcripts in A549 and H1299 cells (**Figure 7F**). These findings suggested that CTNNBIP1 might be downstream of RBM10. RBM10 positively regulates CTNNBIP1, and its expression is regulated by RBM10 at the protein levels**.** In line with these results, we also conducted rescue experiments. In A549 and H1299 cells with stable overexpression of RBM10, si-CTNNBIP1 was transiently transfected to down-regulate the expression of CTNNBIP1. The wound healing assay indicated that CTNNBIP1 knockdown significantly increased cell migration in stable RBM10 overexpression A549 and H1299 cells (**Figure 7G-I**). Moreover, Similar with the wound healing assay, Transwell invasion and migration assay also showed the same results (**Figure 7J-M**). In addition, using colony formation assay, we also observed that RBM10 significantly reduced cell proliferation in A549 cells while silencing CTNNBIP1 reversed this process (**Figure S6A**). Furthermore, Western blotting analysis revealed that silencing CTNNBIP1 suppressed the E-cadherin, whereas enhanced Vimentin and Slug expression in stable RBM10 overexpression cells compared to control cells (**Figure S6B**). Collectively, these data demonstrate that RBM10 interacts with CTNNBIP1 and reduces the protein expression of CTNNBIP1 in LUAD.

### RBM10 inhibits the Wnt/β-catenin pathway by blocking the β-catenin-TCF/LEF interaction

**Figure 8:**
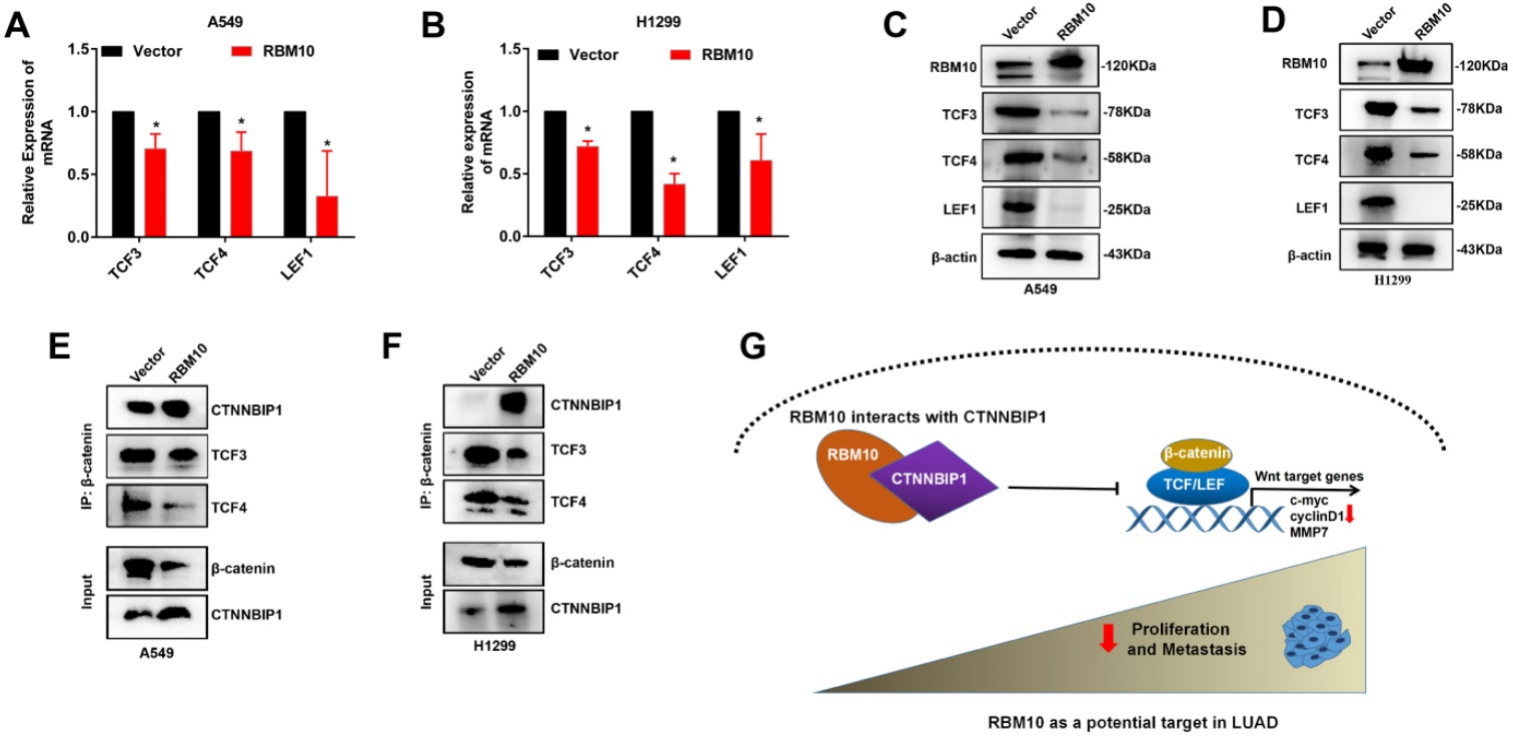
** RBM10 inhibits the Wnt/β-catenin pathway by blocking the β-catenin-TCF/LEF interaction. (A, B)** qRT-PCR results of TCF3, TCF4, LEF1 mRNA levels under RBM10 overexpression, GAPDH was used as loading controls. **(C, D)** Western blot data of TCF3, TCF4, LEF1 protein levels under RBM10 overexpression, β-actin was used as loading controls. **(E, F)** Co-IP was used to test β-catenin interaction analyses using a β-catenin antibody. Western blot showed that upregulation of RBM10 decreased β-catenin-TCF/LEF interaction, whereas promoting CTNNBIP1/β-catenin interaction. **(G)** A schematic diagram of the functional roles of RBM10 in LUAD cells. The results were represented as mean ± SD. All *P < 0.05 All experiments were repeated three times.

The precise molecular mechanisms through which RBM10 suppresses the Wnt/β-catenin pathway activity in LUAD cells were further elucidated**.** The results of Western blotting and qRT-PCR assays showed that upregulation of RBM10 led to low expression levels of TCF3, TCF4, and LEF1 both protein and mRNA (**Figure 8A-D**). Therefore, we performed co-IP assays to further confirm whether RBM10 regulated the Wnt/β-catenin pathway through CTNNBIP1. As shown in **Figure 8E, F**, the results of co-IP showed that RBM10 overexpression markedly inhibited the association of β-catenin with TCF/LEF while enhancing the interaction between β-catenin and CTNNBIP1. In general, these results proved that RBM10 inactivated the Wnt/β-catenin pathway by increasing the inhibitory role of CTNNBIP1 and blocking the β-catenin-TCF/LEF interaction.

## Discussion

Currently, the role of RBM10 in lung adenocarcinoma is controversial 15, 36. In this research, we found that RBM10 was down-regulated in LUAD and low RBM10 expression had a significantly shorter survival time and poor prognosis, thus suggesting that the low RBM10 expression was a marker of poor prognosis in LUAD. A series of functional experiments *in vitro* and *in vivo* were performed, and we confirmed that RBM10 inhibited cell proliferation, metastasis and EMT processes in LUAD. Taken together, these results strongly indicate that RBM10 is involved in LUAD progression as a tumor suppressor.

Aberrant activation of the Wnt/β-catenin pathway can promote EMT, invasion, and metastasis of various cancers 37, including LUAD 38. RBM10 and RBM5 have 50% amino acid homology and share functional similarities 39. It has been suggested that RBM5 functions as an anticancer by inhibiting the Wnt/β-catenin pathway 40, 41. In our study, we first substantiated that RBM10 inhibits the EMT process of lung adenocarcinoma at least partly through negatively regulating the Wnt/β-catenin pathway. Recently, the Wnt/β-catenin pathway has been gradually recognized as a potentially important target for anticancer therapy 21, 42. Preclinical and clinical studies have shown that inhibitors targeting the Wnt/β-catenin pathway, such as Wnt974, LGX818, OMP-18R5 (Vantictumab), OMP-54F28 (ipafricept), and CWP232291, can successfully inhibit tumors progression. Hence, our results may help improve treatment strategies for the selection of LUAD patients who may particularly benefit from agents that selectively target blocking the Wnt/β-catenin pathway.

In this study, our results revealed that RBM10 interacts with CTNNBIP1 and positively regulates its expression in LUAD. Notably, we found that RBM10 altered only the protein but not the mRNA levels of CTNNBIP1. We hypothesized that RBM10 may regulate CTNNBIP1 levels post-transcriptionally. This is similar to the results of a study in colorectal cancer 43. RBM10 can suppress tumor cell growth and proliferation by blocking MDM2-mediated ubiquitination and degradation of p53 43. It has been reported that CTNNBIP1 gene is an antagonist of Wnt signaling 44. By interacting with β-catenin, CTNNBIP1 disrupts the binding of β-catenin with TCF/LEF complex and prevents Wnt/β-catenin pathway activation from inhibiting the progression of LUAD. In this study, we observed that RBM10 overexpression reduced TCF3, TCF4 and LEF1 expression in LUAD cells by western blot. These findings may explain why TCF3, TCF4, and LEF1 expression levels were reduced by RBM10 in the present study. Furthermore, we confirmed that overexpression of RBM10 reduced the interaction between β-catenin and TCF/LEF complex while promoting the interaction between β-catenin and CTNNBIP1 via co-IP.

However, there are limitations to the study. First, some of the conclusions in our study have not been confirmed *in vivo*. Second, this study only analyzed the expression level of RBM10 in LUAD tumor tissues and normal control via bioinformatics analysis, but has not yet analyzed the expression level of RBM10 in clinical specimens of lung adenocarcinoma and the correlation between its expression and clinicopathological features by IHC and requires to be explored in the future. Third, this study preliminarily confirmed that RBM10 interact with CTNNBIP1, while whether RBM10 has a direct interaction with CTNNBIP1 and which domain or sequence participates in the above interaction needs to be fully investigated. In additions, it is well known that RBM10 is an RNA binding protein. And it whether can regulate the stability of CTNNBIP1 mRNA or the splicing processes for CTNNBIP1 mRNA. These are all problems that we need to explore and solve in the future. Despite these limitations, we believe our findings may broaden the understanding of mechanisms involved in LUAD progression and suggest that RBM10 may serve as a potential molecular target for the future development of LUAD therapy.

In summary, the present study revealed a first working model for how RBM10 inhibits LUAD tumor growth and metastasis (**Figure 8G**). Specifically, RBM10 interacts with CTNBBIP1 and downregulates CTNNBIP1 expression, thereby disrupting the interaction between β-catenin and TCF/LEF complex and inactivating the Wnt/β-catenin pathway.

## Supplementary Material

Supplementary figures and tables.

## Abbreviations

CCK-8: Cell Counting Kit 8
EMT: epithelial-mesenchymal transition
LUAD: Lung adenocarcinoma
co-IP: Co-Immunoprecipitation
H&E: hematoxylin and eosin
IHC: Immunohistochemistry
NC: negative control
siRNA: small interference RNA
qRT-PCR: quantitative real-time polymerase chain reaction
