# Supplementary material for: RNA-binding motif protein 10 represses tumor progression through the Wnt/β- catenin pathway in lung adenocarcinoma
Source: Int J Biol Sci. 2022 Jan 1;18(1):124–39. doi: 10.7150/ijbs.63598 (PMC8692145; doi:10.7150/ijbs.63598)
Supplement: Supplementary file 1 — Supplementary figures and tables. [file ijbsv18p0124s1.pdf]

Supplementary Figure Legends

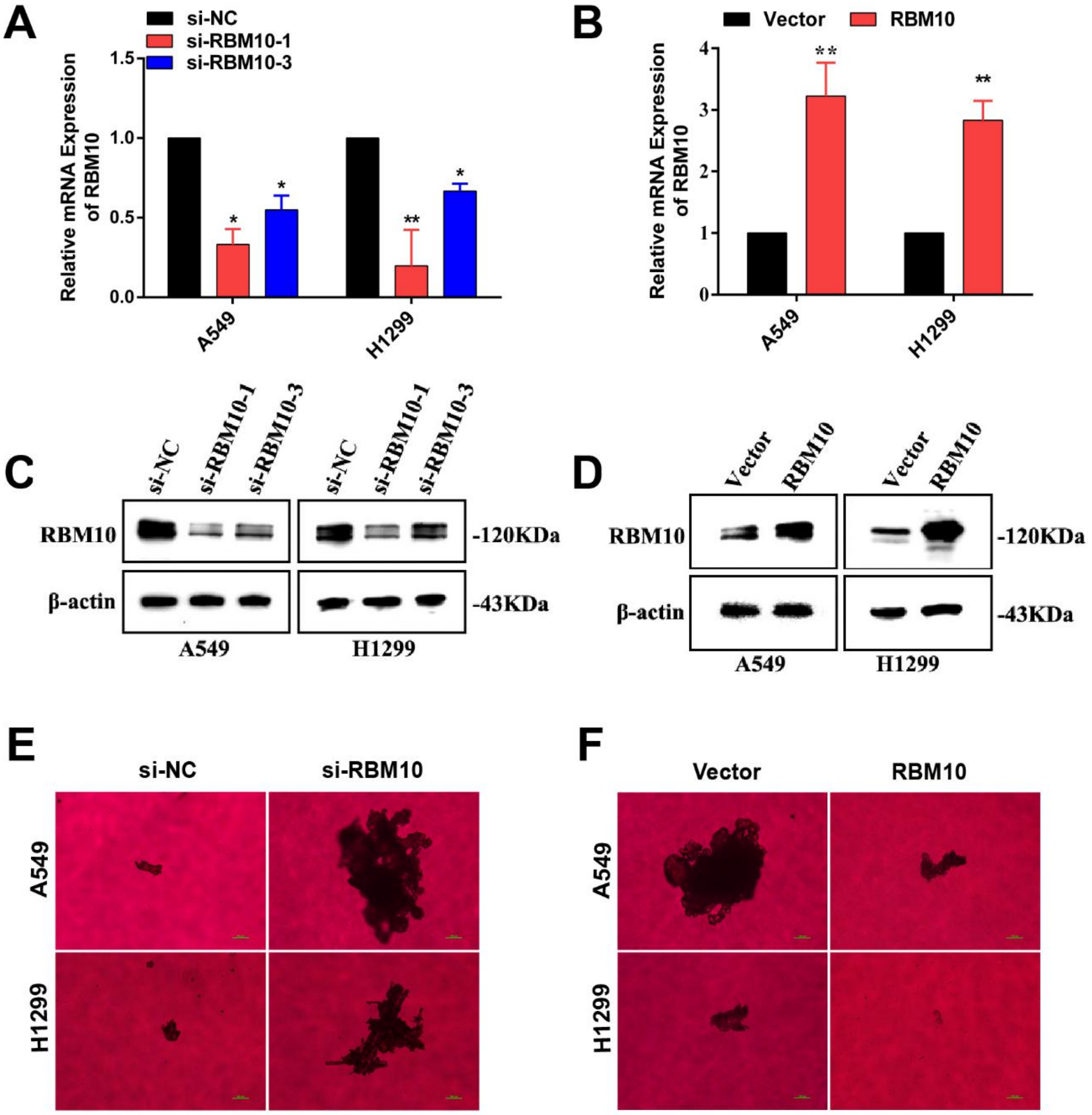

Figure S1 Transfection efficiency of RBM10 and Soft agar colony formation assays

(A~D) Verification of transfection efficiency qRT-PCR and Western blot analysis of RBM10 mRNA and protein expression in A549 or H1299 cell lines transfected with RBM10 si-RNA-1/-3 and si-NC, and the same cell lines infected with RBM10 overexpression lentivirus (RBM10) and negative control (vector). GAPDH was applied as the endogenous control for qRT-PCR, and β-actin was used as a loading control for

western blot assay. The results were represented as mean ± SD. *$P < 0.05$, **$P < 0.01$. **(E, F)** Soft agar colony formation assays using the transfected cells. The scale bar is 100μm. Each experiment was repeated three times.

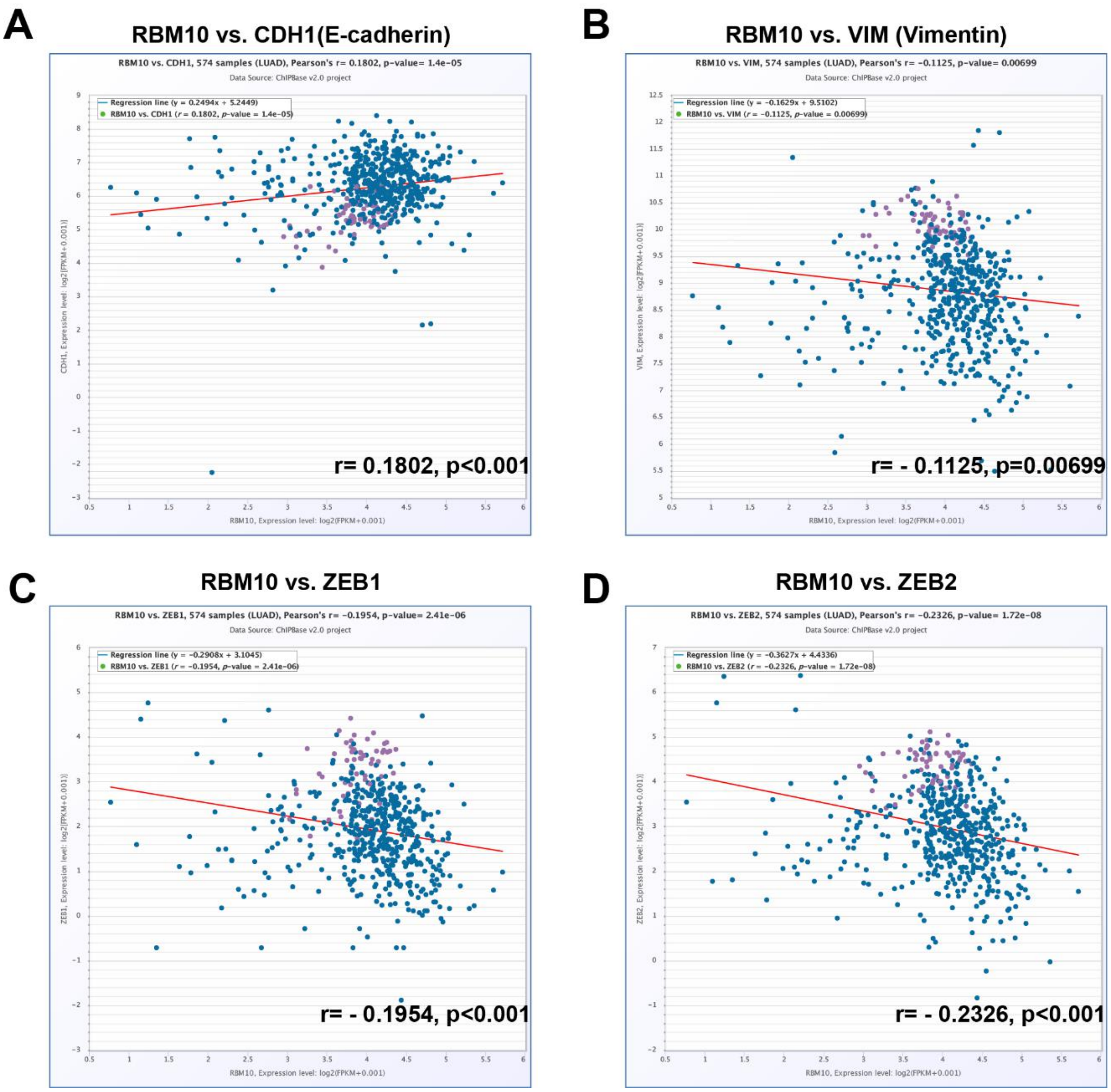

Figure S2 The correlation between RBM10 and EMT markers was analyzed by bioinformatics assays.

The expression relationship between RBM10 and CDH1(E-cadherin), VIM (Vimentin), ZEB1, ZEB2 in LUAD in published database from ChipBase (http://chipbase.sysu.edu.cn/chipbase/).

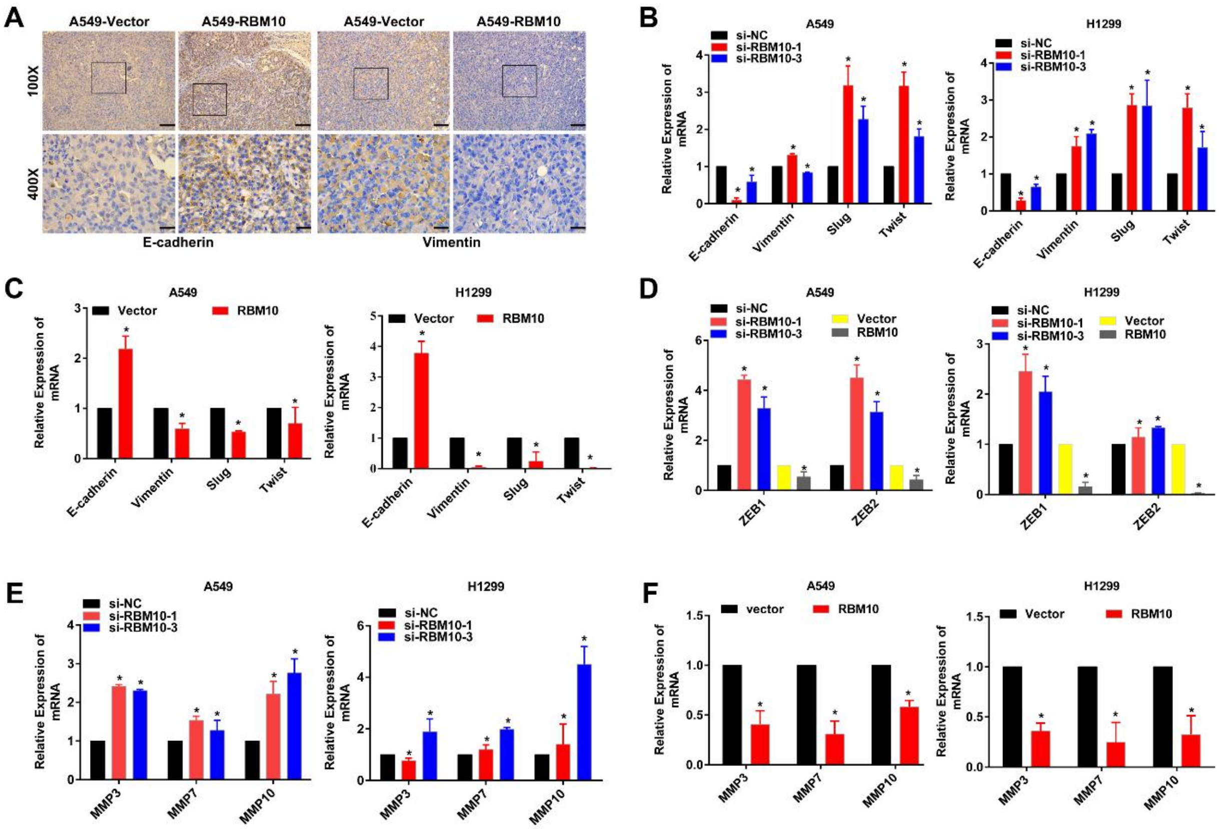

Figure S3 Effect of RBM10 on the protein and mRNA expression of EMT markers

(A) IHC staining of EMT markers (E-cadherin, Vimentin) in xenograft tumors. The scale bar is 100μm. **(B, C)** qRT-PCR results of the EMT markers (E-cadherin, Vimentin, slug, twist) in A549 or H1299 cells with knockdown or overexpressing RBM10. The results were represented as mean ± SD. *P<0.05. **(D~F)** The mRNA levels of ZEB1, ZEB2, MMP3, MMP7 and MMP10 in LUAD cells by qRT-PCR. The results were represented as mean ± SD. *P<0.05, Each experiment was repeated three times.

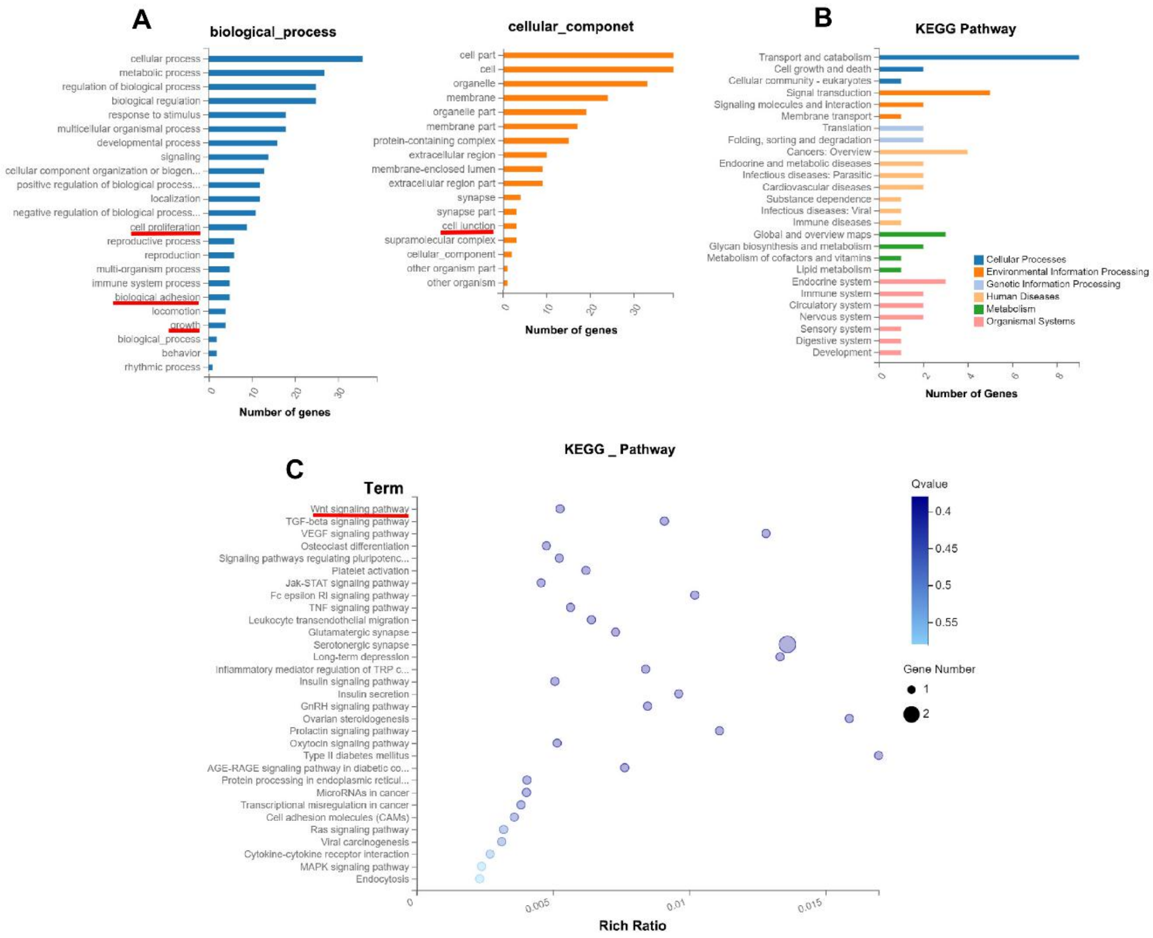

Figure S4 Gene ontology (GO) analysis and KEGG analysis of RBM10-dependent genes.

(A) Gene ontology (GO) analysis involved biological processes (left) such as biological adhesion, cell proliferation and growth, and cellular component (right), including cell junction. **(B)** KEGG analysis was significantly associated with cancer-related functions, including cellular motility, growth and death and etc. **(C)** Pathway analysis of genes that were significantly and differentially expressed in H1299-si-RBM10 cells and control cells using KEGG database.

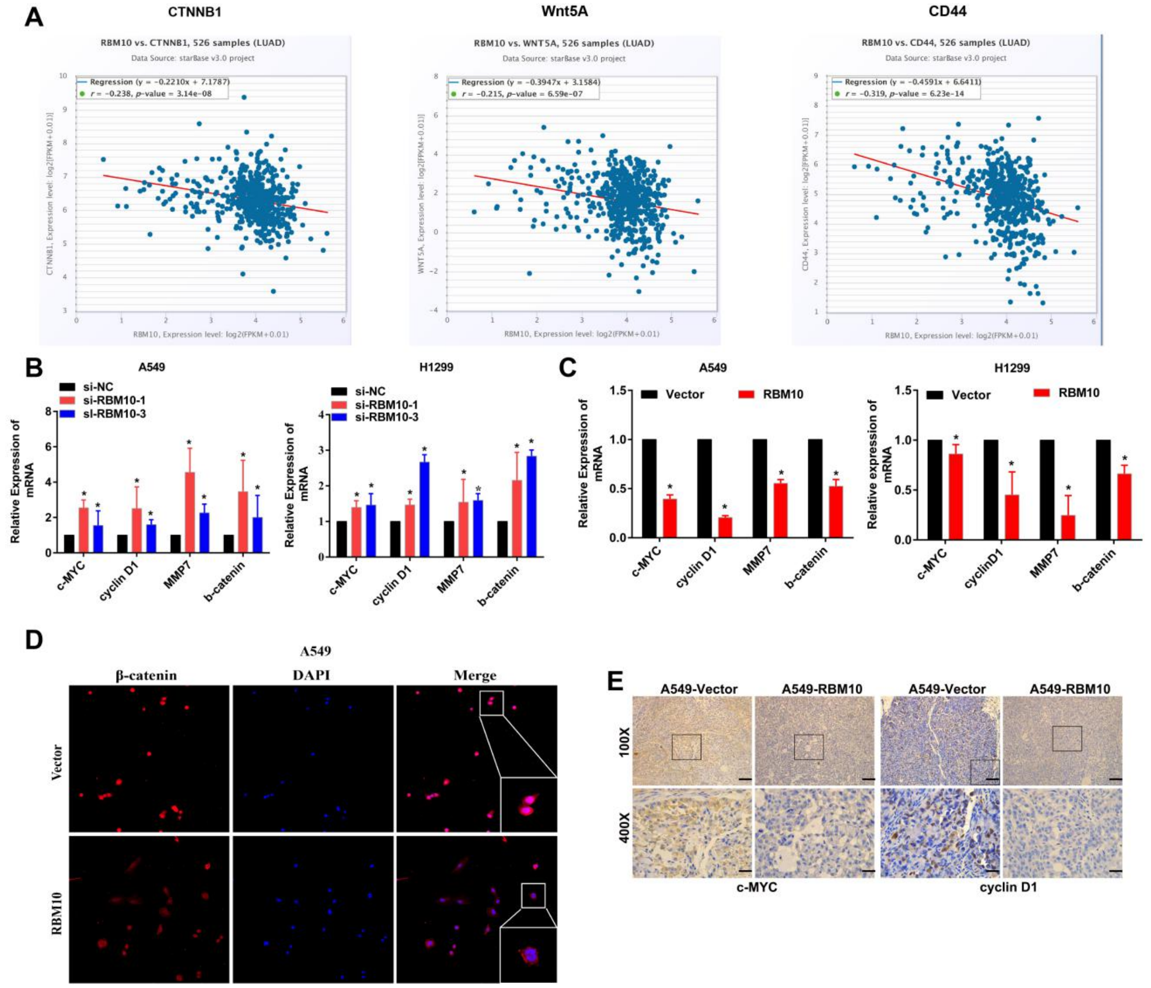

Figure S5 Effect of RBM10 on the Wnt/β-catenin pathway

(A) Negative correlation between RBM10 and the four common of the Wnt/β-catenin pathway target genes (CTNNB1 (also called β-catenin), Wnt5a and CD44) in LUAD, analyzed at the StarBase website. **(B, C)** The effect of RBM10 knockdown **(B)** or overexpression **(C)** on the mRNA levels of β-catenin, cyclin-D1, c-MYC and MMP7 in LUAD cells assayed by qRT-PCR. The results were represented as mean ± SD. *P<0.05. **(D)** IF assay results of β-catenin (red) in RBM10-overexpressing A549 cells. Nuclei counter stained with DAPI (blue). **(E)** The expression of c-MYC and cyclinD1 were examined by IHC staining in the xenograft tumor tissues. The scale bar is 100μm.

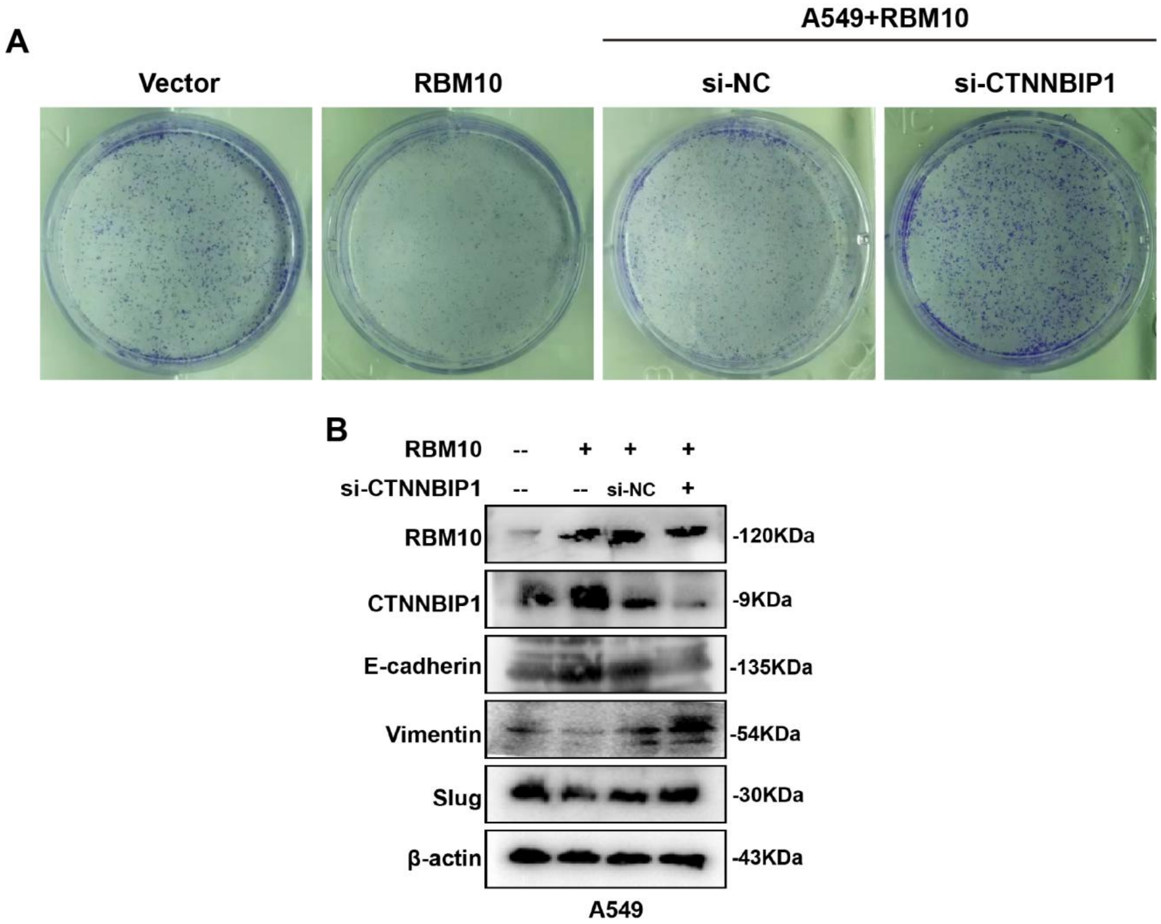

Figure S6 Effect of CTNNBIP1 on cell proliferation and EMT markers (E-cadherin, Vimentin and Slug) in LUAD cells with stable RBM10 overexpression

In A549 and H1299 cells with stable overexpression of RBM10, si-CTNNBIP1 was transiently transfected. (A) clone formation assays; (B) The results of Western blot results of EMT markers (E-cadherin, Vimentin and Slug) after transfection with si-CTNNBIP1 in stable RBM10 overexpression A549 cells. β-actin was used as loading controls. All experiments were repeated three times.

Supplementary Tables

Table S1 Primers sequences for qRT-PCR

GENE	Forward (5’to 3’)	Reverse (5’to 3’)
RBM10	GCACGACTATAGGCATGACAT	AGTCAAACTTGTCTGCTCCA
E-cadherin	GCTGGACCGAGAGAGTTTCC	CAAAATCCAAGCCCGTGGTG
Vimentin	CGGGAGAAATTGCAGGAGGA	AAGGTCAAGACGTGCCAGAG
Slug	TCCTGGTCAAGAAGCATTTCAA	TTGTGGTATGACAGGCATGGA
Twist	GCCAGGTACATCGACTTCCTCT	TCCATCCTCCAGACCGAGAAGG
c-MYC	AGCGACTCTGAGGAGGAAC	TGTGAGGAGGTTTGCTGTG
cyclinD1	GATCAAGTGTGACCCGGACT	CTTGGGGTCCATGTTCTGCT
β-catenin	AAAGCGGCTGTTAGTCACTGG	CGAGTCATTGCATACTGTCCAT
TCF3	CTCGAGAAGAACAGGCCAAG	GGGGCAGGTACTGAACACAT
TCF4	GATGCTCTGGGGAAAGCACT	GTGCCTGCTGAGAGAGATGG
LEF1	AGAACACCCGGATGACGGA	GGCATCATTATGTACCCGGAAT
MMP7	AAATGCCAACAGTTTAGAAGCC	ATTATTTCTATGACGCGGGAGT
MMP3	GGTGTGGAGTTCCTGATGTTGGTC	AGCCTGGAGAATGTGAGTGGAGTC
MMP10	CAGCGGACAAATACTGGAGAT	CTTAGGCTCAACTCCTGGAAAG
CTNNBIP1	GGAAGAGTCCGGAGGAGATG	CTCTGCACCCTGGTCGAT
GAPDH	GACTCATGACCACAGTCCATGC	AGAGGCAGGGATGATGTTCTG

qRT-PCR: Quantitative real-time PCR, RBM10， RNA-binding motif protein 10, CTNNBIP1: β-catenin interacting protein 1

Table S2 Primers antibody for Western blot

Antibody	Manufacturer	Diluted
RBM10	Abcam, Ab72423	1:1000
E-cadherin	Proteintech, 20874-1-AP	1:1000
Vimentin	Proteintech, 10366-1-AP	1:1000
Slug	Cell signaling technology, #9585	1:1000
Twist	Cell signaling technology, #69366	1:1000
N-cadherin	Cell signaling technology, #13116	1:1000
β-catenin	Abcam, Ab32572	1:5000
c-MYC	Abways, CY5150	1:5000
cyclinD1	Abways, CY5404	1:1000
MMP7	Abways, CY1224	1:500
TCF3	Cell signaling technology, #2883	1:1000
TCF4	Cell signaling technology, #2569	1:1000
LEF1	Cell signaling technology, #2203	1:1000
CTNNBIP1	Abcam, Ab129011	1:1000
β-actin	Proteintech, 66009-1-Ig	1:10000

WB: Western blotting assay
